# Oral *Huangqi* Formulae for Stable Chronic Obstructive Pulmonary Disease: A Systematic Review and Meta-Analysis

**DOI:** 10.1155/2013/705315

**Published:** 2013-03-27

**Authors:** Lei Wu, Yuanbin Chen, Yinji Xu, Xinfeng Guo, Xiaoyan Li, Anthony Lin Zhang, Brian H. May, Charlie Changli Xue, Zehuai Wen, Lin Lin

**Affiliations:** ^1^Guangdong Provincial Hospital of Chinese Medicine, Guangzhou 510120, China; ^2^Discipline of Chinese Medicine, School of Health Sciences, RMIT University, Bundoora, VIC 3083, Australia; ^3^National Center for Design Measurement and Evaluation in Clinical Research, Guangzhou University of Chinese Medicine, Guangzhou 510405, China

## Abstract

*Objective*. To evaluate the efficacy and safety of oral *Huangqi* formulae for the treatment of stable COPD. *Methods*. The major databases were searched until September 2010 and supplemented with a manual search. Randomized controlled trials (RCTs) of oral *Huangqi* formulae that reported on lung function, St. George's Respiratory Questionnaire, symptom improvement and/or frequency of exacerbations were extracted by two reviewers. The Cochrane tool was used for the assessment of risk of bias in the included trials. Data were analyzed with RevMan 5.1.2 software. *Results*. 25 RCTs (1,661 participants) were included. Compared with conventional therapy (CT) alone, oral *Huangqi* formulae plus CT increased FEV_1_, and a similar result was found comparing *Huangqi* formulae with no treatment. Improvements in SGRQ total score, COPD-related symptoms and reduction of frequency of exacerbations were found in patients receiving *Huangqi* formulae plus CT compared to those receiving CT alone or CT plus placebo. No serious adverse events were reported. However, there were some methodological inadequacies in the included studies. *Conclusions*. The benefits of *Huangqi* formulae for stable COPD were promising, but its efficacy and safety have not been established due to methodological weakness and possible bias in the reported results. Further rigorously designed studies are warranted.

## 1. Introduction

Chronic obstructive pulmonary disease (COPD) is characterized by airflow limitation and manifests as progressive dyspnea accompanied by deterioration of lung function [1]. It is a major cause of morbidity, disability, and mortality. The World Health Organization (WHO) estimated that COPD ranked fifth in terms of burden of disease worldwide [2]. In China, COPD affects 8.2% of people aged 40 years or older [3]. It was the fourth leading cause of death in cities and the third in rural areas [4].

Studies have shown that pharmacotherapy cannot modify the trend of decline in lung function [5, 6]. Chinese herbal medicine is commonly used for COPD, especially for the stable stage, in China and other Asian countries. Clinical studies suggest that herbal formulae that include *Huangqi *(*Radix Astragalus membranaceus*) were effective for stable COPD [7–31]. It often serves as a principal medicine (i.e., the ingredient provides the principal curative action on the main syndrome or primary symptom [32]) in formulae for COPD. However, the quality of these studies had not been evaluated systematically, and some of the reports for the effects of *Huangqi *were conflicting. Therefore, this systematic review was conducted to evaluate the evidence for the efficacy and safety of oral *Huangqi* for treating stable COPD.

## 2. Methods

### 2.1. Search Strategy

Comprehensive searches were performed for three English language databases and five Chinese databases, which included PubMed (from 1966), Embase (from 1985), Cochrane Central Register of Controlled Trials, Chinese Biomedical Database (CBM, from 1979), China National Knowledge Infrastructure (CNKI, from 1994), VIP medicine information system (VMIS, from 1989), Wanfang (from 1998), and TCM-Online (from 1949), from the inceptions of the databases to September 2010, without language restriction. A manual search was conducted of evidence-based medicine (EBM) reports on Chinese prescriptions by Japan Society for Oriental Medicine, EBM special committee [31]. The focus of the search was randomized controlled trials (RCTs) of oral *Huangqi *formula for stable COPD.

Search terms included chronic obstructive pulmonary disease, chronic bronchitis, emphysema, COPD, chronic obstructive lung disease, chronic obstructive airway disease, chronic airflow obstruction, traditional Chinese medicine, Chinese herbal drugs, complementary and alternative medicine, phytotherapy, herbs, herbal Medicine, Astragalus, *Huangqi*, *Beiqi*, Milkvetch Root, controlled clinical trial, and their synonyms. The literature was screened based on title, abstract, and full text as needed. Full details on the search strategy are described in the appendix.

### 2.2. Study Selection

The inclusion criteria were as follows: (1) RCTs with patients diagnosed with COPD in the stable stage [1, 33], which manifests as dyspnea, cough, and phlegm which remain stable or are rather mild; (2) *Huangqi* formula (taken orally as decoction, pill, powder, or capsule) alone or in combination with conventional therapy compared with placebo, no treatment, or conventional therapy as controls. *Huangqi* serves as a principal medicine, defined as follows: the properties of *Huangqi* are consistent with the main aims of the formula, or the dosage of *Huangqi* is relatively large (more than 15 g). Conventional therapy includes bronchodilators (beta2-agonists, anticholinergics, methylxanthine), corticosteroids, exercise training, smoking cessation, etc [1]. (3) Outcome measurements include spirometric parameters (forced expiratory volume in one second, FEV_1_), St. George's Respiratory Questionnaire (SGRQ) total score, symptom improvement, and/or frequency of exacerbations. The exclusion criteria were (1) trials that included patients with asthma or other non-COPD disorders; (2) test interventions that were combined with other TCM therapies such as acupuncture, acupoint injection; (3) Chinese herbs or other TCM therapies were used in the control group.

### 2.3. Data Extraction and Risk of Bias Assessment

Two authors (Lei Wu and Yuanbin Chen) independently assessed studies based on the inclusion and exclusion criteria. If needed, two other reviewers (Lin Lin and Zehuai Wen) were consulted. Data on the details of study design, participants, interventions, control medicine, outcome measures, numbers of dropouts, and number and nature of any adverse events reported were extracted to a predefined form.

Assessment of symptom improvement was based on the chronic bronchitis section on the Guidance for Clinical Research on New Drugs of TCM [34], where responses were categorized into four levels (symptom control, very good, good, and no effect). The proportion of patient responses for the following symptoms (cough, sputum, dyspnea, and rale) was assessed according to the previous levels.

Two authors (Lei Wu and Yuanbin Chen) independently assessed the risk of bias of the included studies using the Cochrane tool [35]. Any discrepancies in assessment were decided by discussion. Other authors (Zehuai Wen and Xinfeng Guo) were consulted to make the final decision when needed. To verify unclear information on methodology and therapy, attempts were made to contact the authors of the original papers via phone, email, or mail. If the authors were not contactable after 3 times by phone or email, they were sent mail and given one month to reply.

### 2.4. Data Analysis

Data were analyzed by RevMan 5.1.2 (Cochrane Collaboration), and Stata 12.0 software (StataCorp LP, College Station, TX, USA). Dichotomous data were presented as risk ratio (RR) and continuous outcomes as mean difference (MD), with 95% confidence intervals (95% CI). Statistical heterogeneity was assessed by Cochrane's Q test. If the analysis showed low heterogeneity (*P* ≥ 0.10 and *I* ^2^ ≤ 50%), data were synthesized using a fixed-effects model. Otherwise, a random-effects model was applied. Subgroup analyses were performed if sufficient numbers of RCTs were available and sensitivity analyses were undertaken as required. Publication bias was assessed by funnel plot analysis if the group included more than five trials [36]. Egger's test was conducted if it was difficult to determine whether the funnel plots were symmetrical.

## 3. Results

### 3.1. Description of Studies

**Figure 1:**
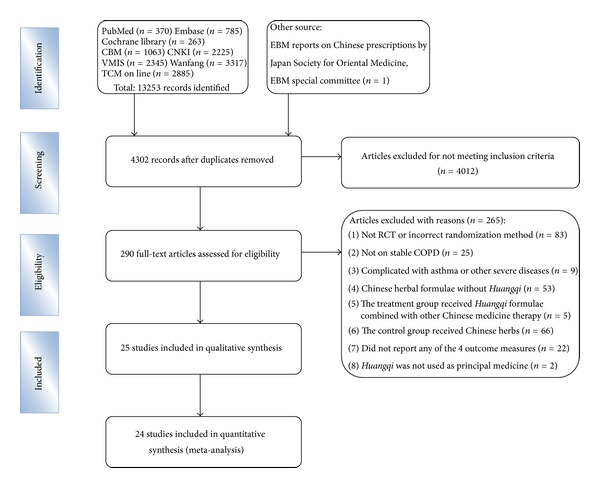
Flow diagram showing the trial selection process for the systematic review.

Of 13,254 potentially relevant citations, 8,952 were excluded as duplications, 4,012 were excluded for not meeting the inclusion criteria after reading the titles and abstracts, and further 265 studies were excluded after reading the full articles and/or contacting the authors. Finally, 25 studies (including 1,661 participants) that met all the selection criteria were included (Figure 1).

Of these 25 studies, 24 were conducted in China [7–30] and 1 in Japan [31]. Twenty-two were published and 3 were dissertations [20, 22, 26]. All patients were diagnosed as stable COPD according to the Global Initiative for Chronic Obstructive Lung Disease (GOLD) or guidelines issued by Committee of Respiratory Disease, Chinese Medical Association. Disease severity in the included trials ranged from mild to very severe COPD. The duration of patients' COPD ranged from 4 to 30 years.

Nineteen studies compared *Huangqi* formulae plus conventional therapy with conventional therapy [7, 9–11, 14–19, 22, 24–31]. Two studies compared *Huangqi* formulae plus conventional therapy with placebo plus conventional therapy [12, 21]. Three studies compared *Huangqi *formulae with no treatment [8, 13, 23]. One study compared a *Huangqi* formula with conventional therapy [20]. The duration of treatment varied from 2 weeks to 6 months. One trial reported a 1-year follow-up period [16].

Five studies indicated that *Huangqi* was used as the principal medicine [12, 17, 18, 20, 26]. In the twenty remaining studies, we considered *Huangqi* to be the principal medicinal when its properties were consistent with the main aims of the formulae or its dosage was more than 15 g, even when the authors did not indicate that it was used as a principal medicinal. The characteristics of the included studies are summarized in Tables 1 and 2.

### 3.2. Risk of Bias of the Included Studies

**Figure 2:**
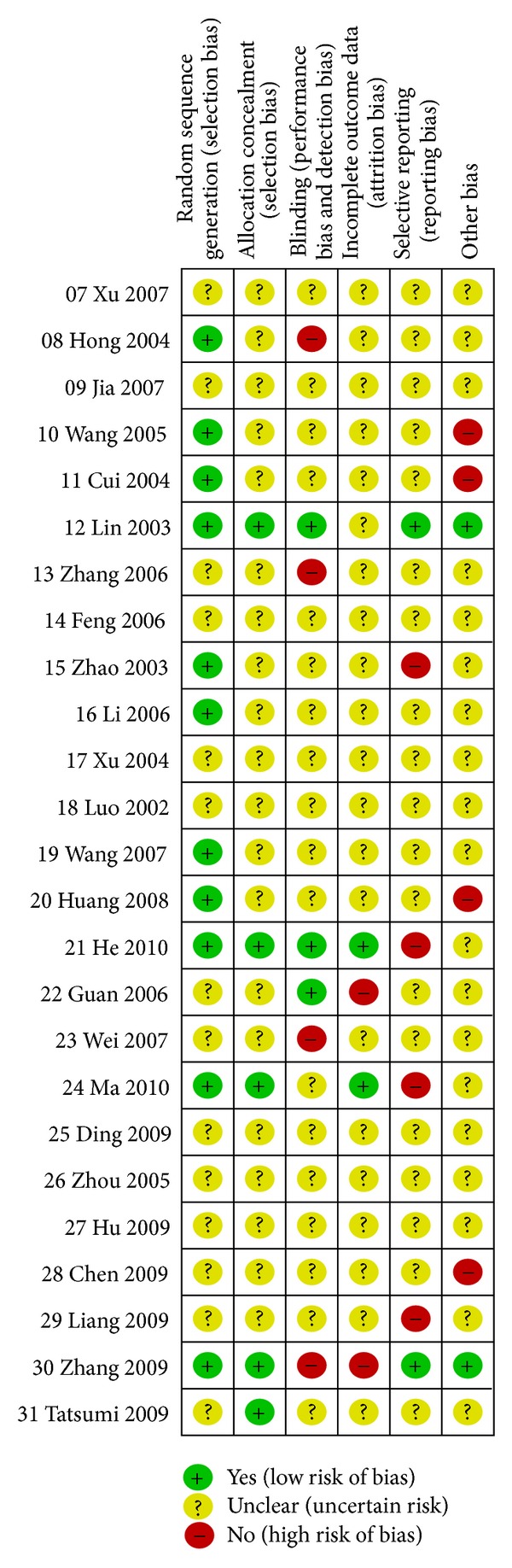
Summary of assessment of risk of bias for each included study.

After attempts at verification by contacting the authors of the original papers through phone, e-mail, or mail, 14 of the included trials still lacked a detailed description of the method of randomization, and 19 trials did not indicate whether blinding was applied. Only two trials described the method of generation of randomization sequence, allocation concealment, double blinding, and placebo manufacturing method. Two trials reported dropouts; one of them explained the reason was loss of contact with the participants, and the other did not provide reasons. No trials reported whether they had used intention-to-treat analysis. Selective outcome reporting was judged as low risk of bias in 2 trials, uncertain risk in 19 trials, and high risk in 4 trials. No study had a registered or published protocol; so, judgment was based on the outcome measures specified in the method section of the study and was informed by discussion with the author when possible. Most of the included trials lacked a sample size calculation (Figure 2).

### 3.3. Publication Bias

**Figure 3:**
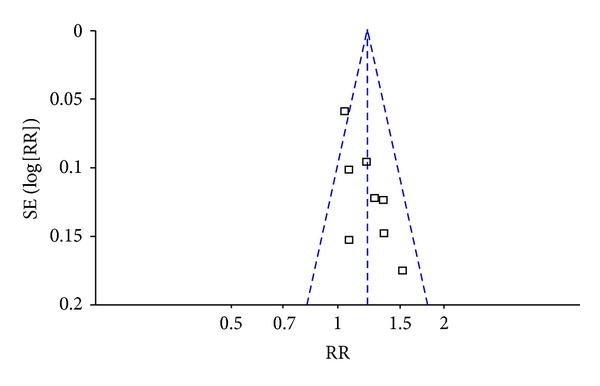
Funnel plot of publication bias using symptom improvement.

The number of studies reporting SGRQ scores and the frequency of exacerbations was less than five; so, a funnel plot was not applicable. The characteristics of studies reporting FEV_1_ were different, and the number of trials in each subgroup was less than five; so, funnel plots were also not applicable. For symptom improvement, the symmetry of the funnel plot was not clear; so, Egger's test was conducted. It indicated that the effect of publication bias was not significant (*t* = 1.39, 95% CI, −0.69 to 2.48, *P* = 0.215) (Figure 3).

### 3.4. Outcome Measures

#### 3.4.1. Spirometric Parameters

**Figure 4:**
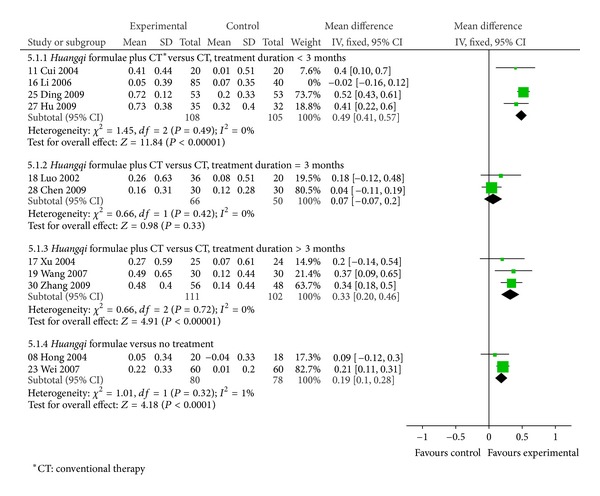
Forest plot of *Huangqi* formulae plus conventional therapy versus conventional therapy, or Huangqi formulae versus no treatment in patients with stable COPD: change in FEV_1_ (L).

Subgroup analyses were performed according the type of comparison (Figure 4). Subgroup 1 compared *Huangqi* formulae plus conventional therapy with conventional therapy alone [11, 16–19, 25, 27, 28, 30]. FEV_1_ increased 0.27 L (95% CI: 0.11 to 0.43) based on 9 trials, but there was high heterogeneity in this subgroup. So, further analyses were performed according to duration of treatment as follows: more than, equal to, or less than 3 months. The former 2 categories had low heterogeneity. There was a significant difference in FEV_1_ in the trials which had durations of more than 3 months (MD 0.33, 95% CI: 0.20 to 0.46) [17, 19, 30] and no significant difference in those of 3 months duration (MD 0.07, 95% CI: −0.07 to 0.20) [18, 28]. For trials of less than 3 months [11, 16, 25, 27], FEV_1_ increased (MD 0.36, 95% CI: 0.29 to 0.43), but there was heterogeneity. So, sensitivity analysis was conducted. It produced low heterogeneity with a significant difference (MD 0.49, 95% CI: 0.41 to 0.57) when one trial [16] was removed because the age distribution of participants differed from the other trials.

Subgroup 2 compared *Huangqi* formulae with no treatment [8, 23]. FEV_1_ increased significantly by 0.19 L based on 2 trails (95% CI: 0.10 to 0.28).

#### 3.4.2. Quality of Life

**Figure 5:**
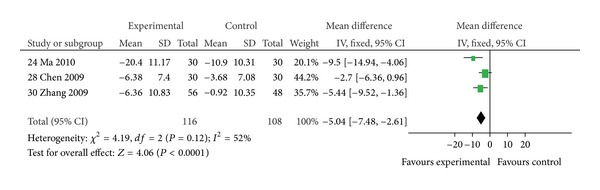
Comparison of *Huangqi* formulae plus conventional therapy versus conventional therapy alone in patients with stable COPD: change in SGRQ total scores.

Patients receiving *Huangqi* formulae plus conventional therapy showed a significantly greater reduction in SGRQ total score (MD −5.04, 95% CI: −7.48 to −2.61) than those receiving conventional therapy alone, based on 3 trials [24, 28, 30] (Figure 5).

#### 3.4.3. Symptom Improvement

**Figure 6:**
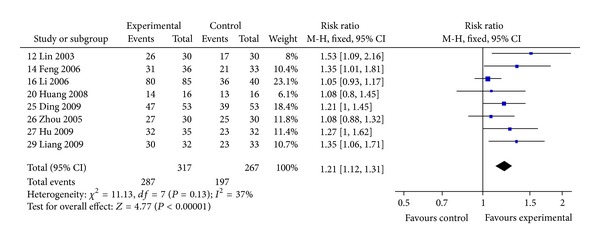
Comparison of *Huangqi *formulae plus conventional therapy versus conventional therapy alone in patients with stable COPD: overall symptom improvement (defined as “good” or above).

Patients receiving *Huangqi* formulae plus conventional therapy were more likely to show improvements in COPD-related symptoms (RR: 1.21, 95% CI: 1.12 to 1.31) when compared with those receiving conventional therapy alone or conventional therapy plus placebo, based on 8 trials [12, 14, 16, 20, 25–27, 29] (Figure 6).

#### 3.4.4. Frequency of Exacerbations

**Figure 7:**
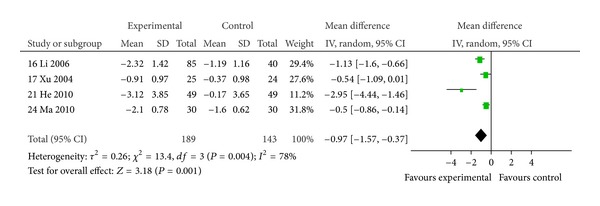
Comparison of *Huangqi* formulae plus conventional therapy versus placebo plus conventional therapy or conventional alone in patients with stable COPD: change in frequency of exacerbations.

Four trials reported frequency of exacerbations at pre- and posttreatment [16, 17, 21, 24]. Compared with placebo plus conventional therapy or conventional therapy alone, the frequency of exacerbations in patients receiving *Huangqi* formulae plus conventional therapy was significantly reduced (MD −0.97, 95% CI: −1.57 to −0.37), but the heterogeneity was high (Figure 7). This appeared due to variation between trials in terms of the control interventions used, duration, and trial quality. Overall, the better result appeared to be of He et al., 2010 [21].

### 3.5. Adverse Events

Six trials reported that no adverse events occurred [7, 8, 11, 21, 26, 31]. One trial (60 participants) reported hoarseness in the control group treated by salmeterol/fluticasone [28], but there was no causality assessment for this adverse event. The other trials did not report adverse events; so, there was insufficient data to assess whether the combination of *Huangqi* formulae plus conventional therapy affected the adverse event rate.

## 4. Discussion

This systematic review includes 24 RCTs conducted in China and 1 RCT in Japan. All RCTs employed the herb *Huangqi* as a principal herb in the herbal formulae used in the test arms, and all studies only included patients assessed as suffering stable COPD. The comparators were mostly conventional therapy, but this was variable and was not clearly specified in some studies. This is likely to have been a source of heterogeneity in the meta-analyses, but it also reflects usual care since patients with stable COPD would typically receive a variety of conventional therapies which may be varied according to response and individual need. Eight studies were of six months, while the others were of shorter treatment duration and only one had a followup at one year; so, the results can only be considered relevant to the relatively short-term management of COPD.

The meta-analysis results indicate that the use of *Huangqi* formulae could significantly improve lung function measured as FEV_1_ when compared with no treatment based on two studies, and it produced an additional improvement when combined with conventional therapies based on 9 studies, four of which were of over three months duration. The incidence of exacerbations also appeared to decline when *Huangqi* formulae were combined with conventional therapies. Improvements in quality of life based on SGRQ were evident, but this was based on only three studies. Also, these *Huangqi*-containing formulae appeared to be well tolerated, even when combined with conventional medications, since seven studies reported that no adverse events were noted.

All the included trials demonstrated at least some methodological deficiencies leading to potential risks of bias. Only eleven provided evidence of adequate randomization procedures, and only three were effectively blinded to participants and investigators. Consequently, the results should be interpreted with caution. Therefore, the potential benefits of oral *Huangqi* formulae for stable COPD need to be further appraised through trials that employ rigorous methodology and include adequate assessment of the safety profiles of the interventions. In addition, we found that the reporting of trial methods and procedures was frequently unclear and insufficient. Therefore, we suggest that all reports of RCTs published in China should be required to comply with the CONSORT statement [37] and the publication of protocols should be encouraged.

There has been increasing interest in complementary and alternative medicine (CAM) for the treatment of COPD, especially the use of Chinese herbal medicines [38, 39]. A recent cross-sectional study in Australia suggested that nearly one in five (17.3%) individuals with moderate to severe COPD had used some form of herbal preparation [39]. Therefore, reviews of the state of the evidence base are essential.

From viewpoint of TCM, patients with stable COPD usually manifest with Qi-deficiency syndrome [40]. One of the characteristics of Qi-deficiency is that the patient easily suffers from colds which commonly lead to acute exacerbations of COPD. *Huangqi *is one of the principal herbs used for reinforcing Qi. It has been widely used for preventing and alleviating common colds; so, its clinical use in COPD is predicated on a putative benefit in preventing colds and reducing COPD exacerbations.

From the experimental perspective, one line of research into *Huangqi* has focused on its effects on inflammation. The main pathological characteristic of COPD is chronic airway inflammation involving a number of proinflammatory mediators and cytokines. Also, oxidative stress is increased in COPD, which amplifies inflammation and may result in corticosteroid resistance. An invivo study suggests that *Huangqi* may reduce inflammatory infiltration and inhibit the inflammatory response in the airway through downregulating the expression of TNF-*α* and IL-8 [41]. Flavonoids in *Huangqi* may protect the erythrocyte membrane from attack by free radicals and appear to eliminate free radicals [42]. One study has shown that airway inflammation induced by cigarette smoke was reversed by astragaloside IV (AST IV), another active constituent of *Huangqi*, in a dose-dependent fashion. This effect appeared due to its anti-inflammatory and antioxidant properties, including NF-*κ*B inactivation [43]. Other studies suggested that AST IV possesses anti-inflammatory and immune regulation activity and can be used for preventing asthma attacks [44]. The previous pharmacological properties of *Huangqi* may at least partially explain the clinical benefits reported by the studies included in this paper.

An earlier paper [45] evaluated the effects of a diverse range of herbal medicines in COPD, and a subsequent paper narrowed the focus to ginseng and ginseng-containing formulae [46]. The strengths of this paper are it focuses only on formulae that share the same principal ingredient and there is experimental evidence that supports the application of this herb as a modulator of inflammation.

The main limitations to this paper are the potential sources of bias due to methodological defects and inadequacies in reporting. Although publication bias was not a major issue, the previous issues potentially lead to overreporting of positive results, selective reporting of outcome measures, and underreporting of adverse events. Also, the use of a diversity of conventional therapies as comparators makes it difficult to assess the magnitude of any effects and to interpret their clinical significance.

Therefore, the potential benefits of oral *Huangqi* formulae for stable COPD evident in this paper need to be further appraised through suitably powered clinical trials that employ standardized conventional therapies as comparators over a sufficient period to determine whether any effects are of sufficient clinical relevance to warrant modification to current best practice for the management of stable COPD.

## 5. Conclusions 

Oral *Huangqi* formulae appear beneficial in terms of improving lung function, quality of life, and symptoms and in reducing the incidence of exacerbations for patients with stable COPD, but these apparent benefits require further appraisal through higher quality trials that strictly adhere to methodological principles and procedures.

## Figures and Tables

**Table 1:** Characteristics of included studies.

First author, year [Ref]	Location	No. of participants (R/A)	Age Mean ± SD (years)	Severity of COPD	COPD History Mean ± SD (years)
Xu 2007 [7]	China	T: 30/30 C: 30/30	T: 67 ± 19.31 C: 64 ± 20.97	T: II: 12, III: 18 C: II: 13, III: 17	NR
Hong 2004 [8]	China	T: 20/20 C: 18/18	T: 67.70 ± 5.68 C: 66.89 ± 5.57	T: II: 5, III: 15 C: II: 4, III: 14	T: 15.05 ± 5.54 C: 14.33 ± 7.28
Jia 2007 [9]	China	T: 30/30 C: 25/25	T: 61.6 ± 6.1 C: 63.2 ± 5.3	T: I: 5, II: 18, III: 7 C: I: 3, II: 17, III: 5	T: 15.25 ± 4.01 C: 13.12 ± 3.38
Wang 2005 [10]	China	T: 20/20 C: 20/20	T: 59.4 ± 7.5 C: 60.9 ± 7.9	T: II A: 8, II B: 9, III: 3 C: II A: 9, II B: 9, III: 2	T: 11.2 ± 4.1 C: 11.8 ± 4.5
Cui 2004 [11]	China	T: 20/20 C: 20/20	T: 62.5 C: 61.5	NR	NR
Lin 2003 [12]	China	T: 30/30 C: 30/30	T: 62 C: 60.5	NR	T: 16 ± NR C: 15.4 ± NR
Zhang 2006 [13]	China	T: 24/24 C: 22/22	T: 66.70 ± 6.60 C: 67.89 ± 4.57	T: II: 5, III: 19 C: II: 4, III: 18	T: 14.05 ± 6.54 C: 15.33 ± 5.0
Feng 2006 [14]	China	T: 36/36 C: 33/33	48–73	NR	NR
Zhao 2003 [15]	China	T: 15/15 C: 15/15	T: 51.71 ± 8.10 C: 48.87 ± 7.73	NR	NR
Li 2006 [16]	China	T: 85/85 C: 40/40	T: 53 C: 49	NR	T: 22 ± NR C: 25 ± NR
Xu 2004 [17]	China	T: 25/25 C: 24/24	T: 63 ± 6.4 C: 62.9 ± 7.0	NR	T: 17.7 ± 9.5 C: 16.9 ± 12.3
Luo 2002 [18]	China	T: 36/36 C: 20/20	T: 63.86 ± 12.56 C: 63.90 ± 12.56	NR	T: 20.36 ± 11.21 C: 19.70 ± 10.00
Wang 2007 [19]	China	T: 30/30 C: 30/30	T: 62.8 C: 60.5	NR	T: 15 ± NR C: 18 ± NR
Huang 2008 [20]	China	T: 16/16 C: 16/16	T: 74.75 ± 6.17 C: 73.81 ± 3.71	T: II: 8, III: 8 C: II: 8, III: 8	NR
He 2010 [21]	China	T: 49/49 C: 49/49	75 ± 5.8	NR	11.25 ± 5.86
Guan 2006 [22]	China	T: 37/30 C: 37/30	T: 65.5 ± 8.21 C: 65.10 ± 12.42	T: 0:7, I: 4, II: 4, III: 15 C: 0:4, I: 5, II: 11, III: 10	NR
Wei 2007 [23]	China	T: 60/60 C: 60/60	Not reported	NR	NR
Ma 2010 [24]	China	T: 30/30 C: 30/30	Not reported	I to II	NR
Ding 2009 [25]	China	T: 53/53 C: 53/53	T: 61.23 ± 3.65 C: 62.16 ± 3.03	T: I: 35, II: 18 C: I: 31, II: 22	T: 17.21 ± 3.02 C: 16.79 ± 2.41
Zhou 2005 [26]	China	T: 30/30 C: 30/30	T: 63.63 ± 7.77 C: 64.83 ± 7.37	NR	NR
Hu, 2009 [27]	China	T: 35/35 C: 32/32	T: 64.7 ± 7.5 C: 63.2 ± 5.4	NR	NR
Chen 2009 [28]	China	T: 30/30 C: 30/30	70.1	NR	NR
Liang 2009 [29]	China	T: 32/32 C: 33/33	T: 69.73 C: 70.69	T: I: 0, II: 13, III: 12, IV: 7 C: I: 0, II: 15, III: 12, IV: 6	T: 17.32 ± NR C: 17.02 ± NR
Zhang 2009 [30]	China	T: 60/56 C: 60/48	68.4	NR	NR
Tatsumi 2002 [31]	Japan	T: 34/34 C: 37/37	Not reported	II to III	NR

T: treatment; C: control; NR: not reported; R: number of subjects randomized; A: number of subjects analysed; SD: standard deviation.

**Table 2:** Characteristics of included studies.

First author, year [Ref]	Intervention (ingredients of *Huangqi* formulae)	Control	Duration /followup	Adverse event	Outcome measures			
					Lung function	PESI	SGRQ	FCOPDE
Xu, 2007 [7]	Manzufei decoction (Ginseng, *Huangqi*, Prepared Rehmannia Root, Tatarian Aster Root, White Mulberry Root-Bark, Chinese Caterpillar Fungus, Chinese Magnoliavine Fruit, Liquorice Root, Salvia Root, Peach Seed, Earthworm, Dwarf Lilyturf Tuber, English Walnut Seed) + Bronchodilators	Bronchodilators	2 weeks/NR	No	Yes (no FEV1)	No	No	No

Hong, 2004 [8]	Yufeining pills (Ginseng, *Huangqi*, Polygonatum sibiricum, White Atractylodes Rhizome, Human Placenta, English Walnut Seed, Dodder Seed, Asiatic Cornelian Cherry Fruit, Bitter Apricot Seed, Snakegourd Fruit, Thunberg Fritillary Bulb, Salvia Root, Peach seed)	No treatment	6 mths/NR	No	Yes	No	No	No

Jia, 2007 [9]	Yiqihuoxue decoction (*Huangqi*, Earthworm, Figwort Root, Salvia Root, Heterophylly Falsestarwort Root, Chinese Angelica) + Ipratropium bromide	Ipratropium bromide	6 mths/NR	NR	Yes (no FEV1)	No	Yes	Yes (noncontinuous data)

Wang, 2005 [10]	Yifeijianpi decoction (*Huangqi*, Tangshen, White Atractylodes Rhizome, Poria, Divaricate Saposhnikovia Root, Pinellia Tuber, Dried Tangerine Peel, Earthworm, Common Coltsfoot Flower, Liquorice Root) + Hydrochloric acid ammonia bromine tablet	Salbutamol + Hydrochloric acid ammonia bromine tablet	8 weeks/NR	NR	Yes (no FEV1)	No	No	No

Cui, 2004 [11]	Tongfei decoction (Unprocessed Rehmannia Root, Chinese Angelica, *Huangqi*, Salvia Root, Lily Bulb, Dwarf Lilyturf Tuber, Ginseng, Pinellia Tuber, Thunberg Fritillary Bulb, Snakegourd Fruit, Poria, Liquorice Root, Fructus Perillae, Citrus Red) + Ipratropium bromide+ Oxygen therapy	Ipratropium bromide + Oxygen therapy	4 weeks/NR	No	Yes	No	No	No

Lin, 2003 [12]	Jianpiyifei granule (Ginseng, White Atractylodes Rhizome, Poria, Dwarf Lilyturf Tuber, White Mulberry Root-Bark, *Huangqi*) + Conventional treatment	Placebo + Conventional treatment	2 mths/NR	NR	Yes (no FEV1)	Yes	No	No

Zhang, 2006 [13]	Jianpiyifeibushen decoction (*Huangqi*, Tangshen, White Atractylodes Rhizome, Poria, Dwarf Lilyturf Tuber, Coastal Glehnia Root, Malaytea Scurfpea Fruit, Dodder Seed, Glossy Privet Fruit, Tokay Gecko, Bitter Apricot Seed, Snakegourd Fruit, Thunberg Fritillary Bulb, Salvia Root, Sichuan Lovage Rhizome)	No treatment	6 mths/NR	NR	Yes (no FEV1)	No	No	Yes (noncontinuous data)

Feng, 2006 [14]	Jianpibufei decoction (Heterophylly Falsestarwort Root, *Huangqi*, Tokay Gecko, White Atractylodes Rhizome, Largetrifoliolious Bugbane Rhizome, Chinese Thorowax Root, Chinese Angelica, Common Coltsfoot Flower, Dodder Seed, Leech) + Doxofylline tablet + Contracting lip breathing + Oxygen therapy	Doxofylline table + Contracting lip breath + Oxygen therapy	30 days/NR	NR	Yes (no FEV1)	Yes	No	No

Zhao, 2003 [15]	Herbal decoction (*Huangqi*, Cassia Bark, Radix Aconiti Praeparata, Prepared Rehmannia Root, Common Yam Rhizome, Asiatic Cornelian Cherry Fruit, Poria, Oriental Waterplantain Rhizome, Tree Peony Root Bark, Dwarf Lilyturf Tuber, Chinese Magnoliavine Fruit) + Pulmonary rehabilitation	Pulmonary rehabilitation	4 weeks/NR	NR	Yes (no FEV1)	No	No	No

Li, 2006 [16]	Bufeihuoxue Decoction (*Huangqi*, White Atractylodes Rhizome, Common Yam Rhizome, Peach Seed, Salvia Root, Sanqi, Red Peony Root, Safflower, Dried Tangerine Peel, Bitter Apricot Seed, Dwarf Lilyturf Tuber) + Bronchodilators + Expectorants	Bronchodilators + Expectorants	8 weeks/1 year	NR	Yes	Yes	No	Yes

Xu, 2004 [17]	Fufangqiqi decoction (*Huangqi*, Polygonatum Sibiricum, Epimedium Herb, Sanqi) + Ipratropium bromide	Nucleotide and casein oral solution + Ipratropium bromide	6 mths/NR	NR	Yes	No	No	Yes

Luo, 2002 [18]	Baofeidingchuan granule (Tangshen, *Huangqi*, Salvia Root, Chinese Angelica, Unprocessed Rehmannia Root, Dwarf Lilyturf Tuber, Platycodon Root, Liquorice Root, Earthworm, Epimedium Herb, etc) Theophylline controlled release capsules + Carbocisteine + Contracting lip breathing	Theophylline controlled release capsules + Carbocisteine + Contracting lip breathing	3 mths/NR	NR	Yes	No	No	No

Wang, 2007 [19]	Bufeiyishen capsule (Tangshen, *Huangqi*, White Atractylodes Rhizome, Dwarf Lilyturf Tuber, Salvia Root, Chinese Caterpillar Fungus, Prepared Rehmannia Root, etc) + Bronchodilators + Mucus lytic agent + Antioxidants + Oxygen therapy	Bronchodilators + Mucus lytic agent+ Antioxidants + Oxygen therapy	6 mths/NR	NR	Yes	No	No	No

Huang, 2008 [20]	Butihuatan decoction (*Huangqi*, Heterophylly Falsestarwort Root, Cassia Bark, Chinese Angelica, Salvia Root, Fructus Perillae, Radish Seed, Pepperweed Seed, Leaf of Leatherleaf Mahonia)	Salbutamol + Theophylline + Hydrochloric acid ammonia bromine tablet + *α*-Chymo- trypsin	2 mths/NR	NR	Yes (no FEV1)	Yes	No	No

He, 2010 [21]	Buzhongyiqi granule (Ginseng, *Huangqi*, White Atractylodes Rhizome, Chinese Angelica, Dried Tangerine Peel, Divaricate Saposhnikovia Root, Largetrifoliolious Bugbane Rhizome, Poria, Fragrant Solomonseal Rhizome, Coastal Glehnia Root) + Theophylline	Placebo + Theophylline	6 mths/NR	No	Yes (no FEV1)	No	No	Yes

Guan, 2006 [22]	Feikang granule (*Huangqi*, Leech,Pinellia Tuber, Earthworm,) + Conventional therapy	Conventional therapy	3 mths/NR	NR	Yes (no FEV1)	No	No	No

Wei, 2007 [23]	Yiqihuayu decoction (*Huangqi*, Herba Gynostemmatis, Cairo Morningglory Root or Leaf, Largetrifoliolious Bugbane Rhizome, Platycodon Root, Common Anemarrhena Rhizome, Zedoary Rhizome, Peach Seed)	No treatment	3 mths/NR	NR	Yes	No	No	No

Ma,2010 [24]	Bufei decoction (Tangshen, *Huangqi*, Prepared Rehmannia Root, White Mulberry Root-Bark, Malaytea Scurfpea Fruit, Tokay Gecko, Tatarian Aster Root, Chinese Magnoliavine Fruit, Liquorice Root) + Pulmonary rehabilitation	Pulmonary rehabilitation	12 weeks/NR	NR	Yes (no FEV1)	No	Yes	Yes

Ding, 2009 [25]	Herbal decoction (Tangshen, *Huangqi*, Magnetite, English Walnut Seed, Chinese Magnoliavine Fruit, Tatarian Aster Root, Common Coltsfoot Flower, Fructus Perillae, Citrus Red, Aloeswood, Liquorice Root, Human Placenta, Tokay Gecko) + Theophylline tablet	Theophylline tablet	2 mths/NR	NR	Yes	Yes	No	No

Zhou, 2005 [26]	Feisaitong mixture (*Huangqi*, Coix Seed, Platycodon Root, Radish Seed, Salvia Root, Earthworm, Common Anemarrhena Rhizome) + Salbutamol	Salbutamol	1 mth/NR	No	Yes (no FEV1)	Yes	No	No

Hu, 2009 [27]	Jiajianbufei decoction (Tangshen, *Huangqi*, Figwort Root, Dwarf Lilyturf Tuber, Malaytea Scurfpea Fruit, Morinda Root, Dodder Seed, Stemona Japonica, White Mulberry Root-Bark, Dried Tangerine Peel, Platycodon Root, Salvia Root) + Oxygen therapy + Hydrochloric acid ammonia bromine tablet + Theophylline + Salbutamol	Oxygen therapy + Hydrochloric acid ammonia bromine tablet + Theophylline + Salbutamol	2 mths/NR	NR	Yes	Yes	No	No

Chen, 2009 [28]	Jiaweiqiweiduqi decoction (Asiatic Cornelian Cherry Fruit, Common Yam Rhizome, Prepared Rehmannia Root, Tree Peony Root Bark, Oriental Waterplantain Rhizome, Poria, Chinese Magnoliavine Fruit, *Huangqi*, Tangshen, White Atractylodes Rhizome) + salmeterol/fluticasone	Salmeterol/fluticasone	12 weeks/NR	Yes	Yes	No	Yes	No

Liang, 2009 [29]	Dongping decoction (Chinese Caterpillar Fungus, *Huangqi*, White Atractylodes Rhizome, Divaricate Saposhnikovia Root) + Bronchodilators	Bronchodilators	12 mths/NR	NR	Yes (no FEV1)	Yes	No	No

Zhang, 2009 [30]	Yifeiyangyin decoction (Lily Bulb, Unprocessed Rehmannia Root, Prepared Rehmannia Root, Thunberg Fritillary Bulb, Platycodon Root, Fructus Auranti, Dwarf Lilyturf Tuber, Radix Paeoniae Alba, Chinese Angelica, Coastal Glehnia Root, Common Yam Rhizome, Poria, *Huangqi*, Liquorice Root ) + Conventional treatment	Conventional treatment	6 mths/NR	NR	Yes	No	Yes	No

Tatsumi, 2002 [31]	Buzhongyiqi decoction (*Huangqi*, Ginseng, White Atractylodes Rhizome, Liquorice Root, Chinese Angelica, Dried Tangerine Peel, Largetrifoliolious Bugbane Rhizome, Chinese Thorowax Root, Ginger) + Inhaled bronchodilators, inhaled corticosteroids, or both	Inhaled bronchodilators, ICS, or both	6 mths/NR	No	No	No	Yes	Yes

PESI: percentage of effectiveness of symptom improvement; SGRQ: St. George's Respiratory Questionnaire; FCOPDE: frequency of COPD exacerbation; NR: not reported.

## Appendix

### Search Strategies

*English Databases*

#1: Chronic obstructive pulmonary disease OR COPD OR emphysema OR Chronic obstructive lung disease OR Chronic obstructive airway disease OR emphysema, pulmonary disease OR Airflow obstruction, Chronic OR Chronic Airflow Obstruction OR Pulmonary Emphysema
#2: Traditional Chinese Medicine OR Chinese Traditional Medicine OR Chinese Herbal Drugs OR Chinese Drugs, Plant OR Medicine, Traditional OR Ethnomedicine OR Ethnobotany OR Medicine, Kampo OR TCM OR Alternative Medicine OR Complementary Medicine OR Phytotherapy OR Herbology OR Plants, Medicinal OR Plant Preparations OR Plant Extracts OR Plants, Medicine OR Materia Medica OR Single Prescription OR Herbs OR Chinese Medicine Herb OR Herbal Medicine
#3: Clinical Trial OR clinical study OR biomedical research OR Controlled Trial OR Controlled study OR random control Trial OR random control study OR Multicenter Study OR random allocation OR double-blind OR single-blind OR comparative study OR evaluation study OR follow-up study OR prospective study OR research design OR control group OR placebo control OR dummy control OR blinding OR clinical research OR medical trial OR in vivo study OR case control study OR intervention study
#4: Astragalus OR Milkvetch OR *Huangqi* OR *Beiqi* OR Milkvetch Root
#5: #1 AND #2 AND #3
#6: #1 AND #3 AND #4
#7: #5 OR #6.

*Chinese Databases *(search by using simplified Chinese character)

#1: Man Xing Zu Sai Xing Fei Bing (Chronic obstructive pulmonary disease) OR Man Xing Zu Sai Xing Fei Ji Bing (Chronic obstructive pulmonary disease) OR Man Zu Fei (Chronic obstructive pulmonary disease) OR Man Xing Zu Sai Xing Fei Bu Ji Bing (Chronic obstructive lung disease) OR COPD OR Zu Sai Xing Fei Ji Bing (Obstructive pulmonary disease) OR Zu Sai Xing Fei Bing (Obstructive pulmonary disease) OR Man Xing Zu Sai Xing Fei Qi Zhong (Chronic obstructive pulmonary emphysema) OR Zu Sai Xing Fei Qi Zhong (Obstructive pulmonary emphysema)
#2: Zhong Yi (Traditional Chinese Medicine) OR Guo Yi (Chinese Medicine) OR Chuan Tong Yi Xue (Traditional Medicine) OR Han Fang (Kampo) OR Han Yi (Kampo Medicine) OR Dong Yi (Vietnamese traditional medicine) OR Min Zu Yi Yao (Ethnomedicine) OR Bian Zheng (Syndrome Differentiation) OR Zhi Fa (Therapeutic Method) OR Zhi Ze (Therapeutic Principle) OR Zhong Xi Yi (Chinese and Western medicine) OR Zhong Xi Yi Jie He (Integration of Chinese and Western medicine) OR Zhong Xi Yao (Chinese and Western medicine) OR Zhong Yao (Chinese Medicine) OR Cao Yao (Herbal Medicine) OR Zhong Cheng Yao (Chinese patent medicine) OR Zhi Wu Yao (Phytomedicine) OR Zhen Ci (acupuncture) OR Jiu (moxibustion) OR Jing Luo (meridian/channel) OR Xue Wei (acupoint) OR Shui Zhen (Aqueous acupuncture) OR Er Xue (ear point) OR Er Zhen (ear acupuncture) OR Fang Xue (Bloodletting) OR Ci Xue (pricking blood) OR Ci Luo (collateral pricking) OR Dian Zhen (electronic acupuncture) OR Ba Guan (cupping) OR Tui Na (tuina) OR An Mo (massage) OR Mei Hua Zhen (plum-blossom needle) OR San Leng Zhen (three-edged needling) OR Mai Xian (catgut-embedding) OR Yao Yun (hot medicinal compress) OR Guan Chang (enema)
#3: *Huang Qi* (Milkvetch) OR Bei Qi (Milkvetch) OR Huang Qi (variant of Chinese character, Milkvetch)
#4: #1 AND #2
#5: #1 AND #3
#6: #4 OR #5.

## Abbreviations

AST IV: Astragaloside IV
CAM: Complementary and alternative medicine
CBM: Chinese biomedical
CI: Confidence interval
CNKI: China national knowledge infrastructure
COPD: Chronic obstructive pulmonary disease
EBM: Evidence-based medicine
ETSE-CM: Evaluation of Traditional and Scientific Evidence of Chinese Medicine
FCOPDE: Frequency of COPD exacerbationdatabase
FEV1: Forced expiratory volume in 1 second
ICS: Inhaled corticosteroids
IL-8: Interleukin-8
IRN-TCM: International Research Network for Traditional and Complementary Medicine
ITT: Intention-to-treat
IV: Inverse variance
MD: Mean difference
NF-*κ*B: Nuclear factor-*κ*B
PESI: Percentage of effectiveness of symptom improvement
RR: Risk ratio
RCTs: Randomized controlled trials
SGRQ: St. George's Respiratory Questionnaire
TCM: Traditional Chinese medicine
TNF-*α*: Tumor Necrosis Factor-*α*
VMIS: VIP medicine information system
WHO: World Health Organization.
